# Immunoexpression of CXCL12 and CXCR4 in sporadic and Gorlin-Goltz syndrome-related odontogenic keratocysts

**DOI:** 10.4317/jced.59561

**Published:** 2022-05-01

**Authors:** Wliana-Pontes de Lima, Allany-de Oliveira Andrade, Roberta-Barroso Cavalcante, Renato-Luiz-Maia Nogueira, Pollianna-Muniz Alves, Cassiano-Francisco-Weege Nonaka, Manuel-Antonio Gordón-Núñez

**Affiliations:** 1 Universidade Estadual da Paraíba – UEPB, Department of Dentistry, Campina Grande, PB, Brazil; 2 Universidade de Fortaleza – UNIFOR, Department of Oral Pathology, Fortaleza, CE, Brazil; 3 Universidade Federal do Ceará – UFC, Department of Dental Clinic, Fortaleza, CE, Brazil

## Abstract

**Background:**

Differences in the pathogenesis and biological behavior of sporadic and Gorlin-Goltz syndrome-related odontogenic keratocysts (OKCs) have been reported, but the underlying mechanisms are not fully elucidated. Chemokine CXCL12 and its main receptor CXCR4 regulate important events in the pathogenesis of several lesions.

**Material and Methods:**

This study evaluated the immunoexpression of CXCL12 and CXCR4 in sporadic and syndromic OKCs. Twenty-two sporadic OKCs and 22 syndromic OKCs were subjected to immunohistochemistry. The percentages of cytoplasmic (CXCL12 and CXCR4) and nuclear (CXCR4) staining in epithelial and fibrous capsule cells were determined. The results were analyzed statistically using the nonparametric Mann-Whitney test and Spearman correlation test (*p*<0.05).

**Results:**

Higher cytoplasmic expression of CXCL12 was observed in the epithelial lining and fibrous capsule of sporadic OKCs compared to syndromic OKCs (*p*<0.001). No statistically significant differences in the cytoplasmic expression of CXCR4 were observed between syndromic OKCs and sporadic OKCs (*p*>0.05). Compared to syndromic OKCs, sporadic OKCs exhibited higher nuclear expression of CXCR4 in the epithelial lining and lower immunoexpression in the fibrous capsule (*p*<0.05). In the epithelial lining of syndromic OKCs, positive correlation was observed between cytoplasmic and nuclear expressions of CXCR4 (*p*=0.003). In the fibrous capsule of syndromic OKCs and sporadic OKCs, cytoplasmic and nuclear expressions of CXCR4 were positively correlated (*p*<0.001).

**Conclusions:**

The results suggest a potential participation of CXCL12 and CXCR4 in the development of OKCs. The heterogeneous expression of these proteins in syndromic and sporadic OKCs may reflect differences in their pathogenesis and biological behavior.

** Key words:**Odontogenic keratocyst, CXCL12, CXCR4, Immunohistochemistry.

## Introduction

OKC is possibly associated with Gorlin-Goltz syndrome (4,5). This syndrome is an autosomal dominant inherited disorder characterized by the presence of multiple OKCs, basal cell carcinomas distributed throughout the body, and bone malformations (6). Studies have suggested syndromic OKCs are potentially more aggressive (7), with higher probability of growth and expansion and higher recurrence when compared with sporadic OKCs (4,5).

Numerous proteins, including those related to apoptosis (7), innate immune response (8), glucose transport (5), and bone resorption (4), have been investigated in order to identify the differences in biological behavior between OKCs associated with Gorlin-Goltz syndrome and sporadic OKCs. Even though these investigations have contributed to understanding the pathogenesis of these lesions, the mechanisms underlying these differences between sporadic and syndromic OKCs have not been fully elucidated.

Chemokine CXCL12, also known as stromal cell-derived factor-1 (SDF-1), belongs to the group of chemokines without the Glu-Leu-Arg motif in their N terminus (ELR-negative) (9). This protein can interact with its main receptor (CXCR4) and with the atypical receptor (CXCR7) (10). By interacting with CXCR4, chemokine CXCL12 stimulates the migration of several cell types, including epithelial cells (11), fibroblasts (12), endothelial cells (13), and inflammatory cells (14). Also, the CXCL12-CXCR4 signaling pathway can promote osteoclast recruitment (15) and angiogenesis (16). On the other hand, CXCR7 is an atypical chemokine receptor that is unable to mediate conventional signaling and promote cell migration (17).

Several studies have highlighted the participation of chemokine CXCL12 and receptor CXCR4 in different pathological processes, such as neoplasms (9,16,18), rheumatic diseases (9) and respiratory diseases (9). As for odontogenic cysts, the few available studies have suggested potential participation of CXCL12-CXCR4 signaling pathway in the development of radicular cysts (14,15). Little is known, however, about the expression of these proteins in developmental odontogenic cysts. Therefore, the aim of the present study was to assess the immunoexpression of chemokine CXCL12 and receptor CXCR4 in sporadic OKCs associated with Gorlin-Goltz syndrome, thus contributing to explicating the pathogenesis and biological behavior of odontogenic lesions.

## Material and Methods

The convenience sample with 22 cases of syndromic OKCs and 22 cases of sporadic OKCs was obtained from the archives of the Laboratories of Oral Pathology at the Departments of Dentistry of the Paraíba Estate University (UEPB) and of the Fortaleza University (UNIFOR). This study was approved by the Research Ethics Committee of UEPB (Process no 3.373.526). The histopathological diagnosis of OKCs was made according to the fourth classification of odontogenic cysts by WHO (3).

The patients with Gorlin-Goltz syndrome were diagnosed following the criteria proposed by Evans *et al*. (19), and all of them had multiple OKCs. The patients with sporadic OKCs had single lesions and underwent clinical and radiographic assessments to rule out other 

manifestations of Gorlin-Goltz syndrome. OKCs were obtained from surgical enucleation or biopsy (incisional or excisional), whose paraffin blocks had a sufficient amount of biological material for immunohistochemical analysis. Cases with secondary inflammation or with recurrent OKCs were excluded from the study.

After the specimen was fixed in 10% formalin and embedded in paraffin, 3-μm sections were obtained and mounted on glass slides prepared with an organosilane-based adhesive. The tissue sections were deparaffinized, rehydrated, and antigen retrieval was done using citrate buffer, pH 6.0, Steamer, 90oC, 30 minutes. Thereafter, the sections were immersed in 3% hydrogen peroxide to block endogenous peroxidase activity. After incubation with anti-CXCL12 (clone 159, Santa Cruz Biotechnology, Dallas, TX, dilution 1:50, incubation for 18 hours) and anti-CXCR4 (clone 12G5, Santa Cruz Biotechnology, Dallas, TX, dilution 1:50, incubation for 60 min) polyclonal primary antibodies, the sections were washed in Tris-HCl buffer and treated with a dextran polymer mixture (Immunohistoprobe Plus™, Advanced Biosystems Inc., Redwood, CA, USA). Peroxidase activity was detected by immersion of the sections in diaminobenzidine (Liquid DAB+ substrate system, Dako North America Inc., Carpinteria, CA, USA), producing a brownish reaction product. Finally, the histological sections were counterstained with Mayer’s hematoxylin, dehydrated, and coverslipped. The positive control for CXCL12 and CXCR4 was performed with sections of oral inflammatory fibrous hyperplasia and the negative control consisted of omission of primary antibodies.

Immunoexpression of CXCL12 and CXCR4 was assessed quantitatively under a light microscope (Leica DM 500, Leica Microsystems Vertrieb GmbH, Wetzlar, DE) by a previously trained examiner blinded to the type of OKC. Immunoexpression of antibodies was assessed in the epithelial lining and in the fibrous capsule of the lesions. Anti-CXCL12 cytoplasmic immunostaining and anti-CXCR4 cytoplasmic and nuclear immunostaining were investigated.

CXCL12 and CXCR4 immunoexpression was assessed by adapting the method proposed by Brito *et al*. (20). The areas with larger immunoreactivity to the antibodies were identified at 100× magnification. Subsequently, at 400× magnification, 10 fields of epithelial lining and 10 fields of fibrous capsule, located immediately below the epithelium, were photomicrographed (ICC 50HD, Leica Microsystems Vertrieb GmbH, Wetzlar, DE). Immunopositive and immunonegative cells were counted in each photomicrographed field using the Image Processing and Analysis in Java software (Image J, National Institute of Mental Health, Bethesda, MD, USA). The values obtained for each of these fields were summed and the percentage of cells with nuclear or cytoplasmic positivity was determined relative to the total number of cells.

Immunohistochemical findings were assessed using the IBM SPSS Statistics software (version 20.0; IBM Corp., Armonk, NY, USA). The Shapiro-Wilk test revealed the data were not normally distributed. Thus, the Mann-Whitney nonparametric test was used to compare the medians of the percentages of CXCL12 and CXCR4 immunopositive cells between sporadic and syndromic OKCs. Correlations between CXCL12 and CXCR4 immunoexpression were assessed by the Spearman correlation coefficient. A level of 5% (*p* < 0.05) was set for all statistical tests.

## Results

**Figure 1:**
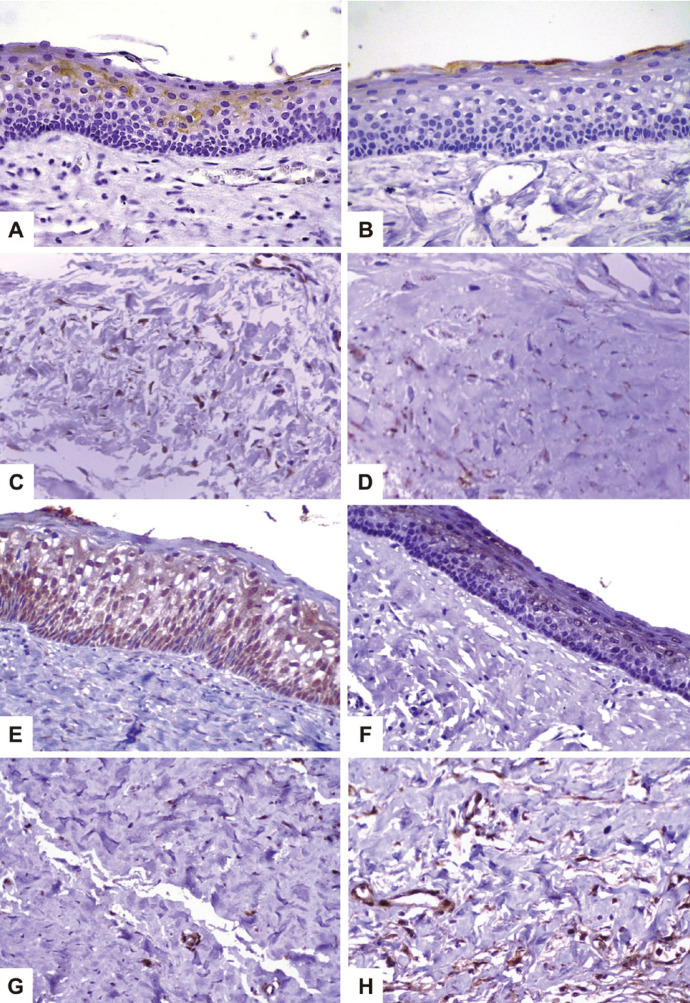
Cytoplasmic expression of CXCL12 in epithelial lining of sporadic (A) and syndromic (B) OKC (Immunohistoprobe Plus™, 400x). Cytoplasmic expression of CXCL12 in cells of the fibrous capsule of sporadic (C) and syndromic (D) OKC (Immunohistoprobe Plus™, 400x). Nuclear expression of CXCR4 in epithelial lining of sporadic OKC (E) (Immunohistoprobe Plus™, 400x). Cytoplasmic and nuclear positivity for CXCR4 in epithelial lining of syndromic OKC (F) (Immunohistoprobe Plus™, 400x). Expression of CXCR4 in cells of the fibrous capsule of sporadic (G) and syndromic (H) OKC (Immunohistoprobe Plus™, 400x).

**Figure 2:**
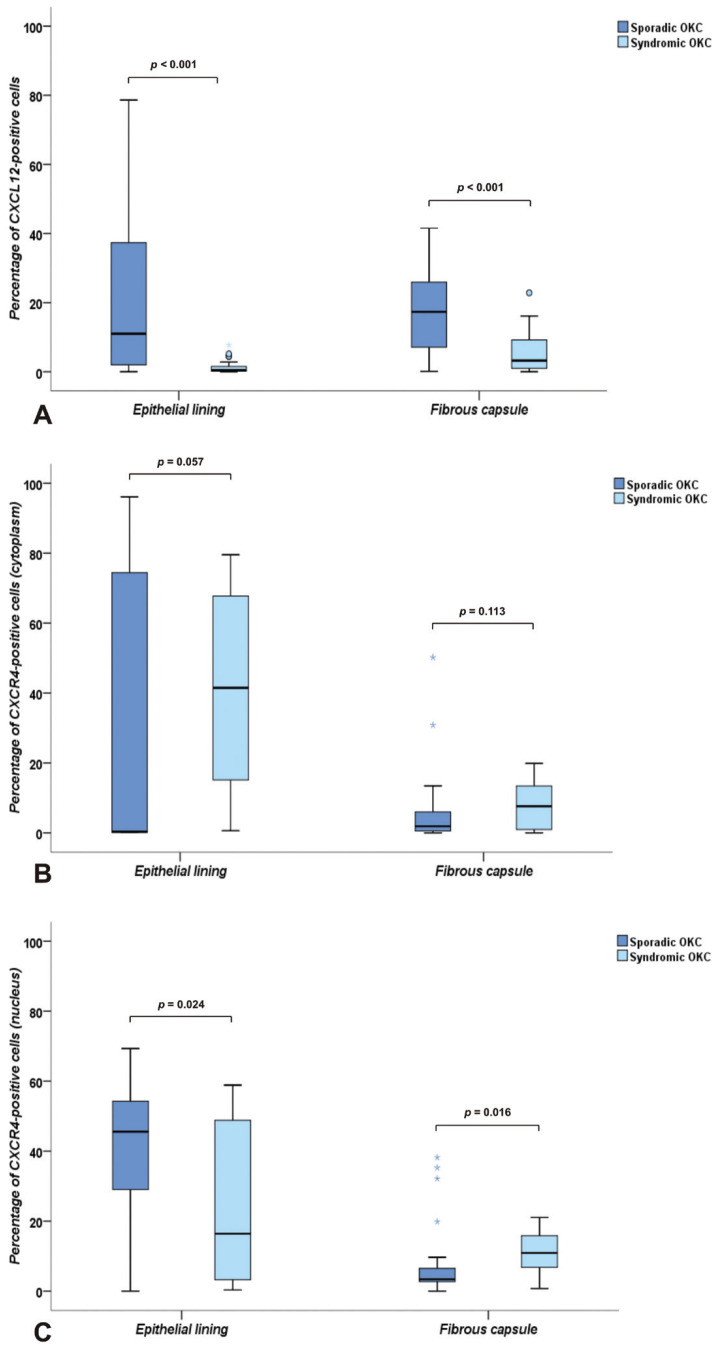
Box plot charts illustrating the percentages of immunopositive cells in sporadic and sybdromic OKCs. Expression of CXCL12 (A). Cytoplasmic expression of CXCR4 (B). Nuclear expression of CXCR4 (C).

Cytoplasmic immunopositivity was predominantly focal in epithelial cells, mainly in suprabasal layers in most of the sporadic (n = 21; 95.4%) and syndromic (n = 20; 90.9%) OKCs

(Fig. 1A,B). Immunopositivity was higher for CXCL12 in the epithelial lining of sporadic OKCs (median: 11.0%; range: 0.0% − 78.6%) when compared to syndromic OKCs (median: 0.5%; range: 0.0% − 7.7%) (*p* < 0.001) (Fig. 2A).

Immunoexpression of CXCL12 in fibrous capsule cells was observed in all sporadic OKCs and in most of the syndromic OKCs (n = 21; 95.4%) (Fig. 1A,B). The immunopositivity rates for CXCL12 were significantly lower in syndromic OKCs (median: 3.2%; range: 0.0% – 22.8%) than in sporadic OKCs (median: 17.3%; range: 0.1% – 41.6%) (*p* < 0.001) (Fig. 2A).

The cytoplasmic immunoexpression of CXCR4 was predominantly diffuse in suprabasal cells of the epithelial lining in the whole sample (Fig. 1C,D). The median of the cytoplasmic immunopositivity rates for CXCR4 in the epithelial lining was higher in syndromic OKCs (median: 41.5%; range: 0.6% – 79.6%) than in sporadic OKCs (median: 0.4%; range: 0.1% − 96.1%), but no statistically significant difference was noted (*p* = 0.057) (Fig. 2B).

The nuclear immunopositivity of CXCR4 was predominantly focal in the suprabasal layers of the epithelial lining in all syndromic OKCs and in most of the sporadic OKCs (n = 21; 95.4%) (Fig. 1C,D). Nuclear immunopositivity for CXCR4 was higher in the epithelial lining of sporadic OKCs (median: 45.6%; range: 0.0% – 69.3%) than in syndromic OKCs (median: 16.4%; range: 0.3% – 58.9%) (*p* = 0.024) (Fig. 2C).

The cytoplasmic immunoexpression of CXCR4 in fibrous capsule cells showed a focal pattern, especially in endothelial cells (Fig. 1E,F). Positivity was detected in most of the sporadic OKCs (n = 21; 95.4%) and syndromic OKCs (n = 20; 90.9%). Regarding the percentage of cytoplasmic positivity for CXCR4 in fibrous capsule cells, no statistically significant differences were observed between sporadic OKCs (median: 1.9%; range: 0.0% – 50.2%) and syndromic OKCs (median: 7.6%; range: 0.0% – 19.9%) (*p* = 0.113) (Fig. 2B).

Nuclear immunoexpression of CXCR4 in fibrous capsule cells was predominantly focal, mainly in the endothelial cells of all syndromic OKCs and in most of the sporadic OKCs (n = 21; 95.4%). The median percentage rate of nuclear immunopositivity for CXCR4 in the fibrous capsule was higher in syndromic OKCs (median: 10.9%; range: 0.7% – 21.0%) than in sporadic OKCs (median: 3.4%; range: 0.0% – 38.2%) (*p* = 0.016) (Fig. 2C).

The correlations regarding the immunoexpression of CXCL12 and CXCR4 in syndromic and sporadic OKCs are summarized in  Table 1. In epithelial lining, there was a positive correlation between the cytoplasmic and nuclear immunoexpression of CXCR4 in syndromic OKCs (r = 0.609; *p* = 0.003). In the fibrous capsule, there was a positive correlation between cytoplasmic and nuclear expression of CXCR4, both in syndromic OKCs (r = 0.700; *p* < 0.001) and sporadic OKCs (r = 0.694; *p* < 0.001).

**Table 1:**
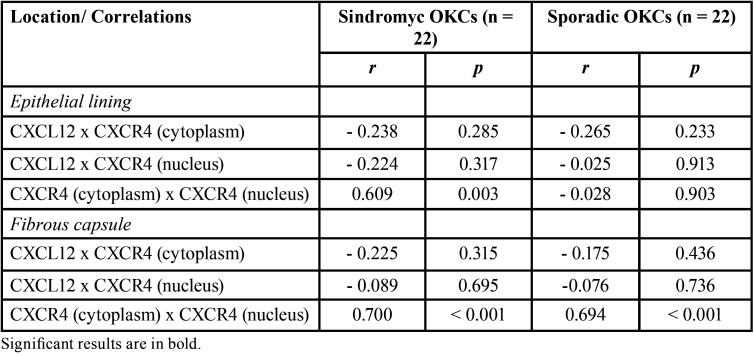
Sample size, Spearman’s correlation coefficient (r) and statistical significance (p) of the immunoexpressions of CXCL12 and CXCR4 in the epithelial lining and fibrous capsule of syndromic and sporadic OKCs.

## Discussion

Chemokine CXCL12 and its receptor CXCR4 participate in several physiologic and pathologic processes (9,16,18), but there is a lack of information about the role of these proteins in the pathogenesis of odontogenic cysts, as the available data are restricted to radicular cysts (14,15). The findings of the present study indicate potential participation of CXCL12 and CXCR4 in the development of OKCs and they also suggest heterogeneous expression of these proteins may demonstrate differences in the pathogenesis and biological behavior of sporadic and syndromic OKCs.

Numerous studies have demonstrated the interaction between CXCL12 and CXCR4 can stimulate important biological processes, such as cell migration (11,14,13,21), maintenance of the integrity of the epithelial lining (11), and differentiation of fibroblasts into myofibroblasts (22). In addition to these functions, findings of research studies on radicular cysts suggest CXCL12-CXCR4 signaling can contribute to osteoclast differentiation (15) and persistent recruitment of immune cells (14). Based on this information, it is possible to check the complexity of CXCL12 and CXCR4 functions and to suggest some potential participation of this signaling pathway in the development of OKCs.

Particularly, the findings of the present study revealed predominance of immunoreactivity to CXCR4 and CXCL12 in the suprabasal layers of the epithelial lining of syndromic and sporadic OKCs. Given the capacity of the CXCL12-CXCR4 pathway to mediate events related to cell survival (17), these findings seem to indicate the participation of these proteins in maintaining the integrity of the epithelial lining of OKCs and, possibly, in the expansion of these lesions.

In malignant neoplasms, a higher expression of CXCL12 has been associated with more aggressive biological behaviors (18,23). In this study, however, syndromic OKCs revealed lower positivity rates for CXCL12 than sporadic OKCs, both in the epithelial lining and fibrous capsule. These findings suggest lower expression of this chemokine can contribute to a more aggressive biological behavior of OKCs associated with Gorlin-Goltz syndrome. On the other hand, syndromic OKCs exhibit higher cytoplasmic positivity for CXCR4, both in the epithelial lining and fibrous capsule, and also higher nuclear positivity for this chemokine receptor in fibrous capsule cells. Conversely, sporadic OKCs showed higher nuclear positivity for CXCR4 in the epithelial lining. The variable immunoexpression of these proteins in sporadic and syndromic OKCs supports the existence of differences in the pathogenesis of these lesions.

There are atypical chemokine receptors (ACKRs), which do not activate classic G protein-dependent signaling cascades (10,24). Binding of these receptors to chemokines can induce the quick internalization and degradation of these proteins (24). Accordingly, CXCL12 can interact with its alternative receptor, CXCR7, also known as ACKR3, or with heterodimers formed between CXCR4 and CXCR7 (10,25,26). After this interaction, CXCR7 induces intracellular signaling and stimulates the relocalization of β-arrestins, eventually leading to the internalization and degradation of CXCL12 (27). Taking these mechanisms into account, the lower expression of CXCL12 in syndromic OKCs observed in this study could be related to a higher degradation of this chemokine in lesions by way of interactions with CXCR7 or with CXCR4/CXCR7 heterodimers. On the whole, these findings underscore the importance of further investigation into the expression of this chemokine receptor in OKCs.

The fibrous capsule of OKCs showed cytoplasmic immunoexpression of CXCL12 and CXCR4, and nuclear expression of CXCR4 in fibroblasts and epithelial cells. Research has demonstrated CXCL12 is a potent chemoattractant for fibroblasts (13), also playing a role in the differentiation of fibroblasts into myofibroblasts during the progression of some lesions (22). Moreover, several studies highlight the participation of CXCL12 in the modulation of vascular events (9,16,24,28). By interacting with glycosaminoglycans or with CXCR4 and/or CXCR7, CXCL12 stimulated the recruitment, activation, and migration of endothelial cells, promoting angiogenesis (9). These findings, in conjunction with the statistically significant positive correlation between cytoplasmic and nuclear immunoexpression of CXCR4, in fibrous capsule cells of syndromic and sporadic OKCs, indicate probable participation of this receptor in the pathogenesis of these lesions.

Research has shown CXCR4 can also interact with non-canonic ligands, such as high mobility group box 1 (HMGB1) protein, macrophage migration inhibitory factor (MIF), extracellular ubiquitin (eUB), and beta-defensin-3 (29), stimulating biological events related to cell migration, mainly of fibroblasts (30), and cell proliferation (29,30). Based on these findings and on the significant immunoexpression of CXCR4 in OKCs observed in the present study, we suggest a potential interaction of this receptor with non-canonical ligands in these odontogenic cysts. These interactions may be particularly important in syndromic OKCs, which exhibited the lowest positivity rates for CXCL12. Together, these findings underscore the importance of future investigation into the expression of non-canonical CXCR4 ligands, as well as into other chemokine receptors, such as CXCR7 in OKCs.

The findings of the present study revealed that, in sporadic OKCs associated with Gorlin-Goltz syndrome, both in the epithelial lining and fibrous capsule, chemokine CXCL12 and its receptor CXCR4 have variable immunoexpression. The heterogeneous expression of these proteins in sporadic and syndromic OKCs may indicate differences in the pathogenesis and in the behavior of these lesions.
